# 88. Increasing Bioburden of *Candida auris* Body Site Colonization is Associated with Environmental Contamination

**DOI:** 10.1093/ofid/ofac492.013

**Published:** 2022-12-15

**Authors:** Sarah Sansom, Gabrielle M Gussin, Raveena D Singh, Pamela B Bell, Ellen C Benson, Jinal Makhija, Mary Carl Froilan, Raheeb Saavedra, Robert Pedroza, Lahari Thotapalli, Christine Fukuda, Ellen Gough, Stefania Marron Rodriguez, Maria del Mar Villanueva Guzman, Julie A Shimabukuro, Lydia Mikhail, Stephanie R Black, Massimo Pacilli, Hira Adil, Cassiana E Bittencourt, Matt Zahn, Nicholas M Moore, Joe Sexton, Judith Noble-Wang, Meghan Lyman, Alaina Whitton, Michael Schoeny, Michael Y Lin, Susan S Huang, Mary K Hayden

**Affiliations:** Rush University Medical Center, Chicago, Illinois; University of California, Irvine School of Medicine, Division of Infectious Diseases, Irvine, California; University of California, Irvine School of Medicine, Division of Infectious Diseases, Irvine, California; Rush University Medical Center, Chicago, Illinois; Rush University Medical Center, Chicago, Illinois; Rush University Medical Center, Chicago, Illinois; Rush University Medical Center, Chicago, Illinois; University of California, Irvine School of Medicine, Division of Infectious Diseases, Irvine, California; University of California, Irvine School of Medicine, Division of Infectious Diseases, Irvine, California; Rush University Medical Center, Chicago, Illinois; Rush University Medical Center, Chicago, Illinois; Rush University Medical Center, Chicago, Illinois; Rush University Medical Center, Chicago, Illinois; RUSH University Medical Center, Chicago, Illinois; University of California, Irvine Health, Orange, California; Orange County Health Care Agency, Orange County, California; Chicago Department of Public Health, Chicago, Illinois; Chicago Department of Public Health, Chicago, Illinois; CDPH, Chicago, Illinois; University of California, Irvine Health, Orange, California; Orange County Health Care Agency, Orange County, California; Rush University Medical Center, Chicago, Illinois; Centers for Disease Control and Prevention, GA; Centers for Disease Control and Prevention, GA; Centers for Disease Control and Prevention, GA; CDC Foundation, Atlanta, Georgia; Rush University, Chicago, Illinois; Rush University Medical Center, Chicago, Illinois; University of California, Irvine School of Medicine, Irvine, CA; Rush University Medical Center, Chicago, Illinois

## Abstract

**Background:**

Environmental contamination is suspected to play a key role in transmission of *Candida auris* in healthcare facilities. We recently showed that environmental surfaces near *C. auris*-colonized patients are commonly recontaminated within hours after disinfection. Clinical factors contributing to environmental contamination are not well characterized.

**Methods:**

We conducted a multi-regional (Chicago, IL; Irvine, CA) prospective study of environmental contamination associated with *C. auris* colonization at six long-term care facilities (LTCF) and 1 acute-care hospital (ACH). On day of sampling, 5 participant body sites were cultured once, followed by routine daily room cleaning by facility staff, then targeted disinfection of high-touch surfaces with hydrogen peroxide wipes by research staff. Surfaces were cultured for *C. auris* using pre-moistened sponge-sticks and neutralizer immediately pre- and post-disinfection, and 4, 8, and 12 hours post-disinfection. We calculated the odds of surface recontamination after disinfection as a function of body site colonization with *C. auris* using generalized estimating equations to account for clustering among multiple surfaces within timepoints, patients, and facilities. Models included an interaction between facility type and colonization.

**Results:**

*C. auris* was cultured from ≥1 body site in 41 participants (12 ACH and 29 LTCF patients, 205 body sites) on day of sampling. Proportion of body sites colonized did not vary by facility type (**Table**). Although environmental contamination rates were similar prior to disinfection [ACH 38% (n=60 samples) vs LTCF 29%, (n=145 samples), p=0.209)], the proportion of surfaces recontaminated between 4–12 hours after disinfection was higher in ACH vs LTCF (n=574 samples) (**Figure**). Number of body sites colonized with *C. auris* was associated with higher odds of environmental recontamination [ACH: OR 2.16 (95% CI 1.63–2.88), p< 0.001; LTCF: OR 1.40 (95% CI 1.07–1.84), p=0.015; Interaction ACH vs LTCF p< 0.001].

**Conclusion:**

The number of body sites colonized was associated with odds of *C. auris* environmental contamination. Differences in environmental recontamination by facility type may be related to greater provider-patient interactions in ACH as a driving factor.

**Disclosures:**

**Gabrielle M. Gussin, MS**, Medline: Conducted studies in which hospitals and nursing homes received contributed antiseptic and/or environmental cleaning products|Stryker: Conducted clinical studies in which hospitals and nursing homes received contributed antiseptic products|Xttrium Laboratories: Conducted clinical studies in which hospitals and nursing homes received contributed antiseptic products **Raveena D. Singh, MA**, Medline: Conducted studies in which hospitals and nursing homes received contributed antiseptic and/or environmental cleaning products|Stryker: Conducted clinical studies in which hospitals and nursing homes received contributed antiseptic products|Xttrium Laboratories: Conducted clinical studies in which hospitals and nursing homes received contributed antiseptic products **Raheeb Saavedra, AS**, Medline: Conducted studies in which hospitals and nursing homes received contributed antiseptic and/or environmental cleaning products|Stryker: Conducted clinical studies in which hospitals and nursing homes received contributed antiseptic products|Xttrium Laboratories: Conducted clinical studies in which hospitals and nursing homes received contributed antiseptic products **Nicholas M. Moore, PhD, D(ABMM)**, Abbott Molecular: Grant/Research Support|Cepheid: Grant/Research Support **Susan S. Huang, MD, MPH**, Medline: Conducted studies in which hospitals and nursing homes received contributed antiseptic and/or environmental cleaning products|Molnlyke: Conducted clinical studies in which hospitals received contributed antiseptic product|Stryker: Conducted clinical studies in which hospitals and nursing homes received contributed antiseptic products|Xttrium Laboratories: Conducted clinical studies in which hospitals and nursing homes received contributed antiseptic product **Mary K. Hayden, MD**, Sanofi: Member, clinical adjudication panel for an investigational SARS-CoV-2 vaccine.

**Figure:**
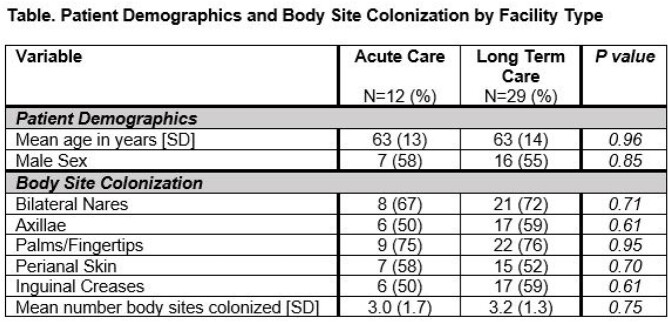

**Figure.**
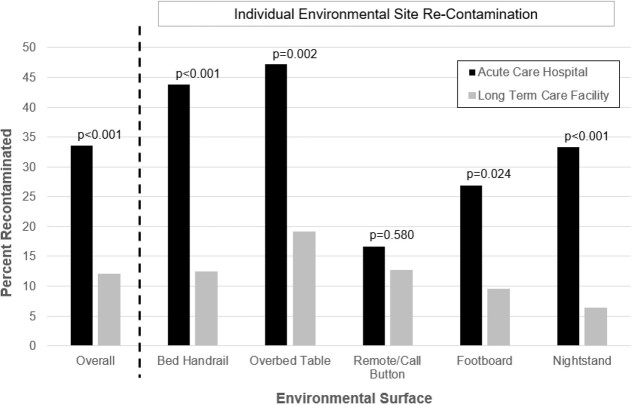
Percent of Environmental Surfaces Recontaminated with C. auris within 12 hours of Cleaning by Facility Type

